# Preclinical *in vivo* evaluation of a gonococcal multivalent vaccine containing antigens identified by CASS

**DOI:** 10.3389/fimmu.2025.1688536

**Published:** 2025-09-22

**Authors:** Shea K. Roe, Bo Zheng, Sunita Gulati, Caroline Genco, Sanjay Ram, Paola Massari

**Affiliations:** ^1^ Department of Immunology, Tufts University School of Medicine, Boston, MA, United States; ^2^ Division of Infectious Diseases and Immunology, University of Massachusetts Chan Medical School, Worcester, MA, United States

**Keywords:** gonorrhea, combination vaccine, bactericidal antibodies, protection, mouse model

## Abstract

**Background:**

*Neisseria gonorrhoeae*, causative agent of the human sexually transmitted infection gonorrhea, is a significant global health concern because of increasing antimicrobial resistance and the lack of an effective vaccine. Recent ecological analyses have shown a reduced incidence of gonorrhea in recipients of detergent-extracted outer membrane vesicle (OMV)-containing meningococcal vaccine, which has contributed to identification of shared, protective antigens. Previously, our group has developed an immunobioinformatics-based pipeline (CASS, Candidate Antigen Selection Strategy) for identification of gonococcal hypothetical proteins expressed during human natural mucosal infections, as novel vaccine candidates.

**Methods:**

In this study, we expanded the immunological characterization of three targets, NGO0690, NGO0948 and Csp (copper storage protein, previously called NGO1701) to include analysis of their efficacy in a mouse model of gonococcal vaginal infection when combined as a trivalent subunit vaccine and adjuvanted with Alum and MPLA.

**Results:**

We reported induction of systemic and mucosal antibody responses, serum bactericidal activity against heterologous *N. gonorrhoeae* strains, and accelerated bacterial clearance in vivo. Immune profiling revealed a balanced Th1/Th2 response, based on IgG antibody subclasses and cytokines. Antigen dose de-escalation experiments in female and male mice showed sustained antibody production against the individual antigens and against whole bacteria. The latter were slightly lower than with the original dose vaccine particularly in male mice, who also exhibited a distinct cytokine pattern and weaker complement-mediated serum bactericidal activity (SBA) titers compared to female mice. These findings underscore the importance of considering sex-based differences in vaccine evaluation. A bivalent vaccine containing only NGO0690 and Csp was still protective *in vivo*, supporting the value of multivalent approaches to address gonococcal antigenic diversity.

**Discussion:**

Overall, our results suggested that the rational design of our multi-antigen subunit vaccines holds translational potential for enhancing broadly protective immune responses and protection against *N. gonorrhoeae*.

## Introduction

*Neisseria gonorrhoeae* is an obligate human pathogen and the causative agent of gonorrhea, a sexually transmitted infection (STI) with an estimated global burden exceeding 82 million cases annually, and in the United States alone, over 600,000 cases were reported in 2023 (1). In men, gonococcal urethral infections are generally symptomatic, but infection in women is frequently asymptomatic; this lack of symptoms can lead to a delayed diagnosis and treatment, with serious reproductive complications including pelvic inflammatory disease, ectopic pregnancy, and infertility (2). Rectal and pharyngeal infections in men who have sex with men (MSM) are also frequently asymptomatic. Unfortunately, natural gonococcal infection induces limited and strain-specific immunity, with scarce memory responses even after repeated exposure (3). In some instances, disseminated gonococcal infection (DGI) can also occur. Concurrent chlamydia infection increases the burden of gonococcal infection (4, 5) and gonorrhea is associated with enhanced transmission of human immunodeficiency virus 1 (HIV-1) infection (6, 7). Furthermore, widespread and increasing antimicrobial resistance (AMR) has been reported for gonorrhea in the last decade. Resistance has emerged even to extended-spectrum cephalosporins, the last FDA-recommended first-line treatment, raising fears of untreatable gonorrhea (8–10). While efforts to identify new treatment options are ongoing, developing gonococcal vaccines is essential for prevention of disease transmission (11).

Vaccine development for gonorrhea has been hampered by antigenic variation, immune evasion, and absence of natural immunity. Earlier approaches with killed whole–cell, pilin or porin subunit, and outer membrane (OM) protein vaccines failed due to genetic variability, and conferred only short-term or strain-specific protection [reviewed in (12)]. A significant breakthrough emerged from retrospective epidemiologic studies indicating that detergent-extracted meningococcal outer membrane vesicle (OMV)-based vaccines (MeNZB and 4CMenB (Bexsero^®^)) conferred partial protection against *Neisseria gonorrhoeae* (13). 4CMenB generated bactericidal antibodies and significantly accelerated clearance of *N. gonorrhoeae* in a mouse model of gonococcal vaginal colonization (14–17). While serum bactericidal activity (SBA) is an established correlate of protective immunity for *N. meningitidis* (18), the correlates of protection against gonorrhea have not been established, but SBA is accepted as *in vitro* surrogate assay in preclinical gonococcal vaccine studies (19). Building on these observations, multiple clinical trials are underway to evaluate the efficacy of 4CMenB against gonococcal infection, and to identify relevant correlates of protection (20–22). *N. meningitidis* and *N. gonorrhoeae* share multiple conserved outer membrane proteins (80–90% genome homology) (23–26), which is likely the cause of the observed cross-protective responses. Gonococcal native OMVs formulated with slow-release IL-12 microspheres and administered either intranasally or intravaginally elicited Th1 responses, suppressed non-protective Th17 pathways, and accelerated clearance of gonococci from mouse vaginas (27, 28). Importantly, protection was abrogated in B cell-deficient mice, underscoring the role of antibodies in vaccine-induced immunity.

Meanwhile, individual gonococcal components also have shown promise in the mouse model and provide an opportunity for developing a subunit vaccine. For example, lipooligosaccharide (LOS) and peptide mimics of conserved LOS epitopes (29, 30), and surface-exposed proteins such as PorB (25), NHBA, MetQ (31) or TbpA/TbpB (32) are a few examples of vaccine targets that have been explored in preclinical studies with various adjuvants (12). Subunit vaccine candidates have shown to be immunogenic in mice, to induce antibodies with bactericidal and/or opsonophagocytic activity, and, in some instances (e.g., MetQ, TbpB, and the LOS mimotope), protection against *N. gonorrhoeae* in a mouse model of gonococcal vaginal challenge. However, these antigens have been identified by growing *N. gonorrhoeae in vitro* in conditions that may not replicate protein expression levels during natural infection in humans. Our group has utilized a novel immunobioinformatics-based pipeline called CASS (Candidate Antigen Selection Strategy) and identified hypothetical proteins that are expressed during natural mucosal infection in men and women and could represent new vaccine antigens (33–35). Thus far, from a pool of 36 gonococcal targets, three antigens have undergone a detailed immunological characterization - NGO0690, NGO0948 (homolog of BamC) and Csp [previously referred to as NGO1701 (36)], either individually (33) or as a combination vaccine with different adjuvants (37). We reported robust immune responses in mice, with serum bactericidal activity against several *N. gonorrhoeae* strains, and recognition of these antigens by sera from human subjects with acute gonococcal infection as well as convalescent subjects (37), supporting the translational potential of these candidates for preclinical studies. Building on these results, and with the premise that a multi-antigen vaccine platform would elicit a broader epitope coverage and more durable response compared to a single antigen vaccine (38–43), we evaluated the efficacy of a NGO0690+NGO0948+Csp combination vaccine with Alum+MPLA as adjuvants (3-Ag vaccine) *in vivo* in a female mouse model of gonococcal vaginal colonization. A multi-antigen approach could also help to address the antigenic variability and overcome immune evasion strategies that have undermined previous gonococcal subunit vaccine attempts (44). In addition, the effect of antigen dose de-escalation was examined on the magnitude of immune responses in both female and male mice, as well as the ability of a simplified two-antigen combination that only included NGO0690 and Csp (2-Ag vaccine), on protection *in vivo* in the mouse model of gonococcal challenge. Our results showed that immunization with the 3-Ag vaccine led to protection against *N. gonorrhoeae in vivo*. Lowering the antigen concentration had a small effect on antibody production in female mice, accompanied by a slight decrease in the serum bactericidal activity titers, while these effects were more pronounced in male mice. Protection was also demonstrated using the 2-Ag vaccine, indicating that excluding the structurally more complex and low immunogenic antigen NGO0948, could still afford protective immune responses *in vivo.* Studies investigating other CASS antigen are currently ongoing to identify additional candidates that could potentiate a multi-antigen vaccine combination, as well as revisiting adjuvants, doses and/or routes of immunization.

## Materials and methods

### Antigens

Recombinant NGO0690, NGO0948 (BamC) and Csp were expressed and purified as previously described (33).

### Immunization of mice

Female and male BALB/c mice (4–6 weeks old) (Jackson Labs, Bar Harbor, ME, USA) were housed, cared for and immunized according to NIH, Tufts University (Protocol number B2024-11) and University of Massachusetts Chan Medical School IACUC (Protocol number 202000074) approved protocols. Groups of mice (n=20) were immunized subcutaneously three times at 2-weeks apart with purified recombinant NGO0690+NGO0948+Csp (10 µg each) combined and adjuvanted with Alum (Imject) (Thermo Fisher Scientific; 1:1 v/v ratio with the antigen mixture) + Monophosphoryl Lipid A (MPLA) (Avanti Lipids; 10 µg/mouse/dose) (37) (referred to as 3-Ag vaccine in the text), or with NGO0690 and Csp (10 µg each) combined and Alum+MPLA as above (referred to as 2-Ag vaccine in the text). Control groups of mice (n = 20) were immunized with Alum+MPLA alone (referred to as Adjuvant in the text). Additional groups of female and male BALB/c mice (n = 5) were immunized with a low-dose 3-Ag vaccine containing 5 µg of each antigen and with Alum+MPLA as above. For all experiments, pre-immune (Pr) sera were collected before immunization, and immune sera two weeks after each immunization (referred to as 1^st^, 2^nd^, 3^rd^ in the Figure legends). An additional aliquot of serum was collected four weeks after the last immunization and prior to infection (pre-challenge, p-c) from the mice undergoing gonococcal challenge described below. Vaginal lavages were collected two weeks after the last immunization. All sera and lavages were stored at -80°C until use.

### Bacterial strains and growth conditions

*N. gonorrhoeae* strains F62 (Pil+/Opa+) and FA1090 were stored as frozen glycerol stocks. Bacteria were grown on GC base agar plates containing 1% (v/v) IsoVitaleX or on chocolate agar at 37°C in a 5% CO_2_ incubator, and in liquid GC broth (GCB) with 1% IsoVitaleX (33). Growth was monitored spectrophotometrically at O.D._600nm_. For some experiments, aliquots of bacteria suspension at O.D._600nm_ > 1 were formalin-killed by incubation with 1% paraformaldehyde for 1h at 4°C, washed and resuspended in PBS.

### Mouse model of gonococcal vaginal challenge

At the end of the immunization schedule, female mice were evaluated to select those in the diestrus phase of the estrous cycle. Groups of 10 mice each were treated with 0.5 mg Premarin (Pfizer) in 200 µL water subcutaneously for 3 days (day −2, 0, and +2 days relative to the challenge (Day 0) to allow for a longer estrus phase and to increase susceptibility to *N. gonorrhoeae* infection as previously described (45). Antibiotics (vancomycin, trimethoprim, and streptomycin, (VTS)) were used to control the mouse vaginal microflora without affecting *N. gonorrhoeae* survival. Mice were challenged intravaginally with *N. gonorrhoeae* FA1090 (2.7 x 10^7^ – 3.1 x 10^7^ colony forming units, CFUs) and infection burden was monitored daily by obtaining vaginal swabs, eluting the content of each swab in 100 µl of normal saline, and plating of serial dilutions of the eluted material onto chocolate agar containing VTS, colistin and neomycin, and counting CFUs after incubation of plates for 24 h at 37°C in 5% CO_2_.

### Mouse antibody ELISA

ELISA plates were coated with purified recombinant NGO0690, NGO0948 or Csp proteins (2 μg/ml) or with formalin-fixed *N. gonorrhoeae* (1–1.5 x 10^8^ bacteria/ml) as previously described (37). Serial dilutions of pooled mouse sera or vaginal lavages were added to measure total IgG, IgG1, IgG2a, IgM or IgA antibodies using AP-conjugated secondary anti-mouse antibodies (Southern Biotech) followed by 1-step PNPP (p-nitrophenyl phosphate) reagent (ThermoFisher Scientific) and spectrophotometric detection at O.D._405nm_. IgG, IgG1, IgG2a and IgM were quantified in µg/ml ± SD using antibody reference standard curves (Southern Biotech) and a linear regression function (33). IgA (O.D._405nm_ values minus the blank) were normalized to the adjuvant alone and expressed as fold-change ± SD. The Th1:Th2 ratio was calculated as IgG2a/IgG1. Sera and vaginal lavages were evaluated in triplicate or quadruplicate.

### Cytokine ELISA

Th1-type cytokines IL-12p70 and IFN-γ, Th2-type cytokines IL-4 and IL-10 and IL-6, TNF-α and IL-1β were measured in pooled mouse sera by ELISA using Opt-EIA kits (BD Biosciences, San Jose, CA, USA) or Invitrogen kit (ThermoFisher Scientific) per the manufacturers’ specifications. Cytokines were expressed in pg/ml ± SD.

### Serum bactericidal activity

SBA was conducted as previously described (37). Briefly, *N. gonorrhoeae* F62 and FA1090 cultures (2–4 x 10^4^ CFU/ml) were incubated in HBSS with 0.15 mM CaCl_2_ and 1 mM MgCl_2_ and 2% BSA for 20 minutes at room temperature with serial dilutions of heat-inactivated mouse sera. Commercially available IgG/IgM-depleted pooled normal human serum (Pel-Freez Biologicals) at a final concentration of 10% (v/v) was added as source of complement. Aliquots of the reaction mixture were plated immediately on GC agar plates in triplicate (Time 0) and after 30 minutes incubation at 37°C (Time 30). Plates were incubated at 37°C in 5% CO_2_ overnight, and survival was determined the CFUs at T30 relative to T0 and expressed as percentage ± SD. Bactericidal titers represent the lowest serum dilution that yielded ≤ 50% survival after 30 minutes. Controls reactions included bacteria alone and bacteria incubated with complement alone.

### Statistical analysis

GraphPad Prism 10 (GraphPad Software, Inc., San Diego, CA) was used to determine statistical significance using unpaired t test and one-way analyses of variance (ANOVA) with Tukey’s multiple comparisons test or with Dunnett’s test. Statistically significant p values are indicated in the figure legends. For mouse challenge experiments, the median time to clearance was evaluated using Kaplan–Meier survival curves, the times to clearance were compared between groups using the Mantel-Cox log-rank test. The mean area under the curve (AUC) of the log_10_ CFU vs. time was computed for each mouse to estimate the bacterial burden over time (cumulative infection) and comparisons between groups were made using Mann-Whitney’s non-parametric test (29).

## Results

### Immunization with NGO0690+NGO0948+Csp and Alum+MPLA as adjuvants (3-Ag vaccine) protects mice from gonococcal vaginal colonization

**Figure 1:**
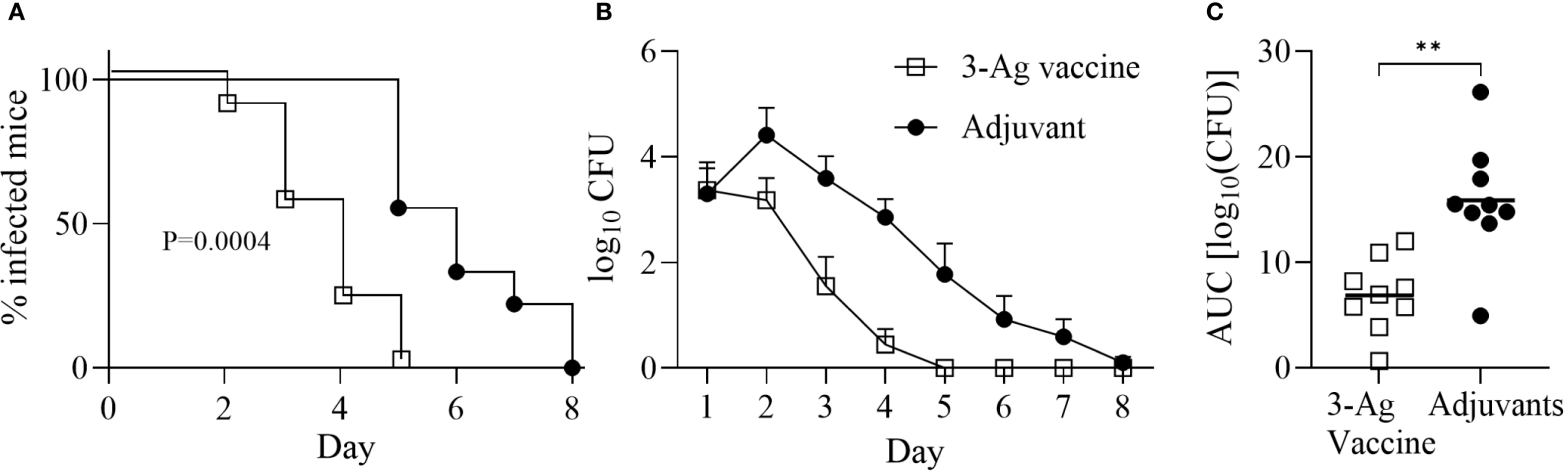
Immunization with NGO0690+NGO0948+Csp and Alum+MPLA (3-Ag vaccine) is protective in a mouse model of gonococcal vaginal colonization. Female BALB/c mice (n = 20 per group) were immunized with the 3-Ag vaccine (10 µg each antigen) (open squares) or with Alum+MPLA alone (black circles). Two weeks after the third immunization, mice in the diestrus phase of the estrous cycle (n = 10 per group) were challenged intravaginally with *N. gonorrhoeae* FA1090 (2.6 × 10^7^ CFU). Vaginal swabs were collected daily and plated to enumerate gonococcal CFUs. **(A)** Time-to-clearance Kaplan–Meier curves (p = 0,0004, groups compared by Mantel-Cox analysis); **(B)** Bacterial burden (log_10_ CFU ± SEM); **(C)** Area under the curve (AUC) (means ± 95% confidence intervals compared across groups by Mann Whitney’s non-parametric test, **p = 0.0019).

We previously characterized immune responses elicited in mice by NGO0690, NGO0948 and Csp [NGO1701 (36)] as individual vaccine targets or in combination, with different adjuvants (33, 37). Here, we evaluated their efficacy as a combination vaccine with Alum+MPLA as adjuvants (3-Ag vaccine) in a mouse model of gonococcal vaginal challenge. Female BALB/c mice were immunized with the 3-Ag vaccine or with Alum+MPLA alone as a control, followed by intravaginal infection with *N. gonorrhoeae* strain FA1090 four weeks after the last immunization. Vaginal swabs were collected daily and plated to enumerate gonococcal CFUs. A significant reduction in the time to bacterial clearance was observed in the 3-Ag vaccine group compared to the adjuvant alone group, shown by the Kaplan–Meier curves (**Figure 1A**, open squares and black circles, respectively). A significant decrease in the number of bacteria recovered was also observed (**Figure 1B**), reflected in the area under the curve (AUC) analysis (**Figure 1C**). These results indicated that the 3-Ag vaccine was protective in a female mouse model of gonococcal colonization.

### Qualitative, quantitative, and functional antibody responses induced by the 3-Ag vaccine

**Figure 2:**
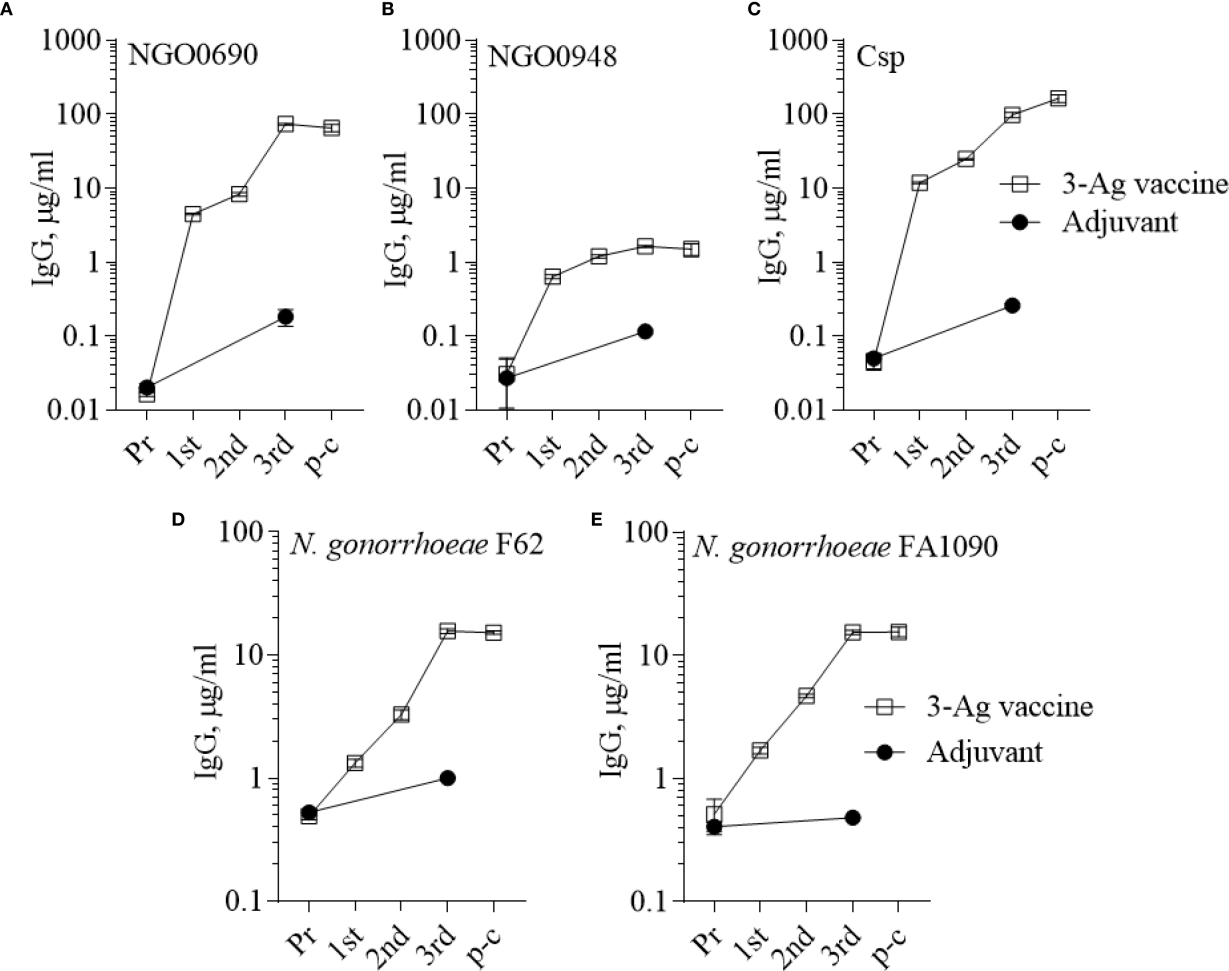
Serum IgG antibodies to the purified antigens and to *N. gonorrhoeae*. Total IgG antibodies measured by ELISA in sera from mice immunized with the 3-Ag vaccine (open squares) or Alum+MPLA alone (closed circles). Sera were tested against **(A)** purified recombinant NGO0690, **(B)** NGO0948 and **(C)** Csp, or **(D)** *N. gonorrhoeae* F62 and **(E)** *N. gonorrhoeae* FA1090 in the mouse preimmune sera (Pr), in sera collected 2 weeks after each immunization (1^st^, 2^nd^ and 3^rd^) and 2 weeks prior to challenge (p-c). Pooled sera were evaluated in triplicate and antibodies were expressed as µg/ml ± SD.

In the current study, mice were immunized with the 3-Ag vaccine three times following a 2-weeks apart schedule, which is different from the prior 3-weeks apart schedule used to characterize the serum antibody responses (37); thus, it was important to evaluate antibody production. IgG levels induced by the 3-Ag vaccine were examined by ELISA against the individual purified antigens or whole *N. gonorrhoeae* using pooled sera aliquots collected throughout the immunization schedule and prior to the challenge. Higher total IgG antibody levels were detected against NGO0690 and Csp than against NGO0948 (**Figures 2A-C**, open circles) consistent with our previous results (37), and these remained elevated prior to the gonococcal challenge (4 weeks after the third immunization, p-c); a small increase in IgG antibodies was also observed in response to immunization with Alum+MPLA alone (**Figures 2A-C**, closed circles). Whole cell ELISA against *N. gonorrhoeae* FA1090 (the strain used in the challenge model) and of *N. gonorrhoeae* F62 (as an additional strain) showed a robust and similar serum IgG antibody response against both strains (**Figures 2D, E**). Analysis of the IgG subclasses confirmed a balanced Th1-Th2 response with a slight Th2 skew (**Supplementary Table S1**) [an IgG2a/IgG1 ratio > 2 indicates a Th1 response, a ratio < 0.5 a Th2-biased response, and between 0.5 and 2, a mixed response (46)]; IgM antibody levels against the antigens and both gonococcal strains (not shown) were also consistent with our previous results (37), also showing presence of non-specific, cross-reactive IgM antibodies in the preimmune sera and in the adjuvant-only group sera. Low serum IgA antibody levels were measured against *N. gonorrhoeae*, although these were about 2-fold higher than with the adjuvant alone (**Supplementary Figure S1A**, dashed bars). However, evaluation of mucosal antibodies showed a 2- to 6-fold increase in secreted IgA over the adjuvant alone group (**Supplementary Figure S1A**, dotted bars) in the vaginal lavages and confirmed induction of IgG antibodies against *N. gonorrhoeae* (**Supplementary Figure S2A**).

**Figure 3:**
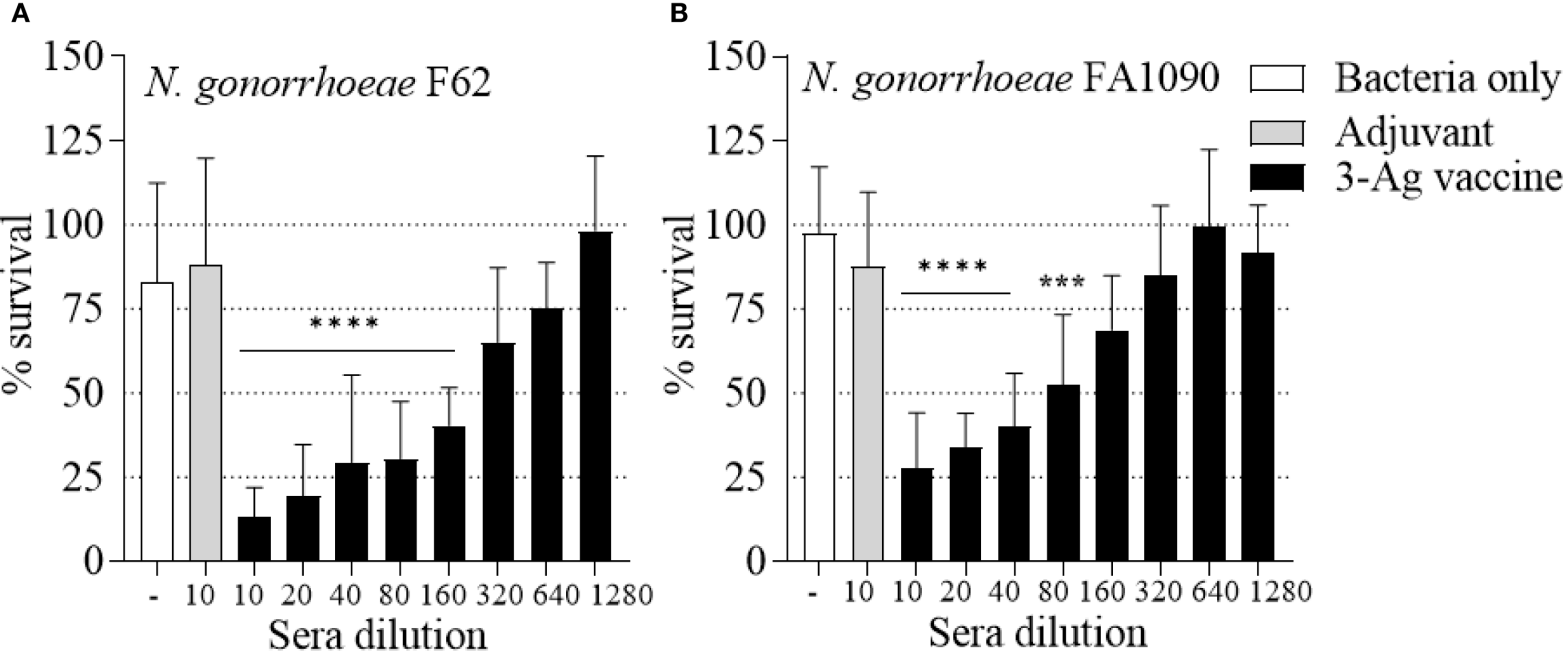
Serum Bactericidal Activity (SBA) of mouse sera elicited by immunization with the 3-Ag vaccine. *N. gonorrhoeae* survival (% CFU at T30/T0 ± SD) in pooled sera from mice immunized with the 3-Ag vaccine (black bars) or Alum+MPLA alone (gray bars). **(A)** *N. gonorrhoeae* F62 and **(B)** *N. gonorrhoeae* FA1090. Sera were evaluated in a minimum of seven repeats and dilutions are indicated on the X-axis. Bacteria alone, white bars. ***p = 0.0005 and ****p < 0.0001 by one-way ANOVA with Dunnett’s multiple comparisons test vs Adjuvant.

As a measure of antibody function, the ability of the immune sera to kill *N. gonorrhoeae* was examined in a complement-dependent bactericidal assay. The 3-Ag vaccine produced sera with killing titers of about 1/160 against *N. gonorrhoeae* F62 (**Figure 3A**, black bars) and of about 1/40 against *N. gonorrhoeae* FA1090 (**Figure 3B**, black bars), also confirming our previous results (37). Differences in killing titers were attributable to the serum-sensitivity profile of these strains [FA1090 is intrinsically more serum-resistant than F62 (45)]. No significant killing was induced by the sera from mice vaccinated with Alum+MPLA alone (**Figures 3A, B**, gray bars). Collectively, these results confirmed that immunization of mice with the 3-Ag vaccine induced robust serum antibody responses and a more modest mucosal antibody response, and that the immune sera had complement-dependent serum bactericidal activity.

### Serum cytokine responses

**Figure 4:**
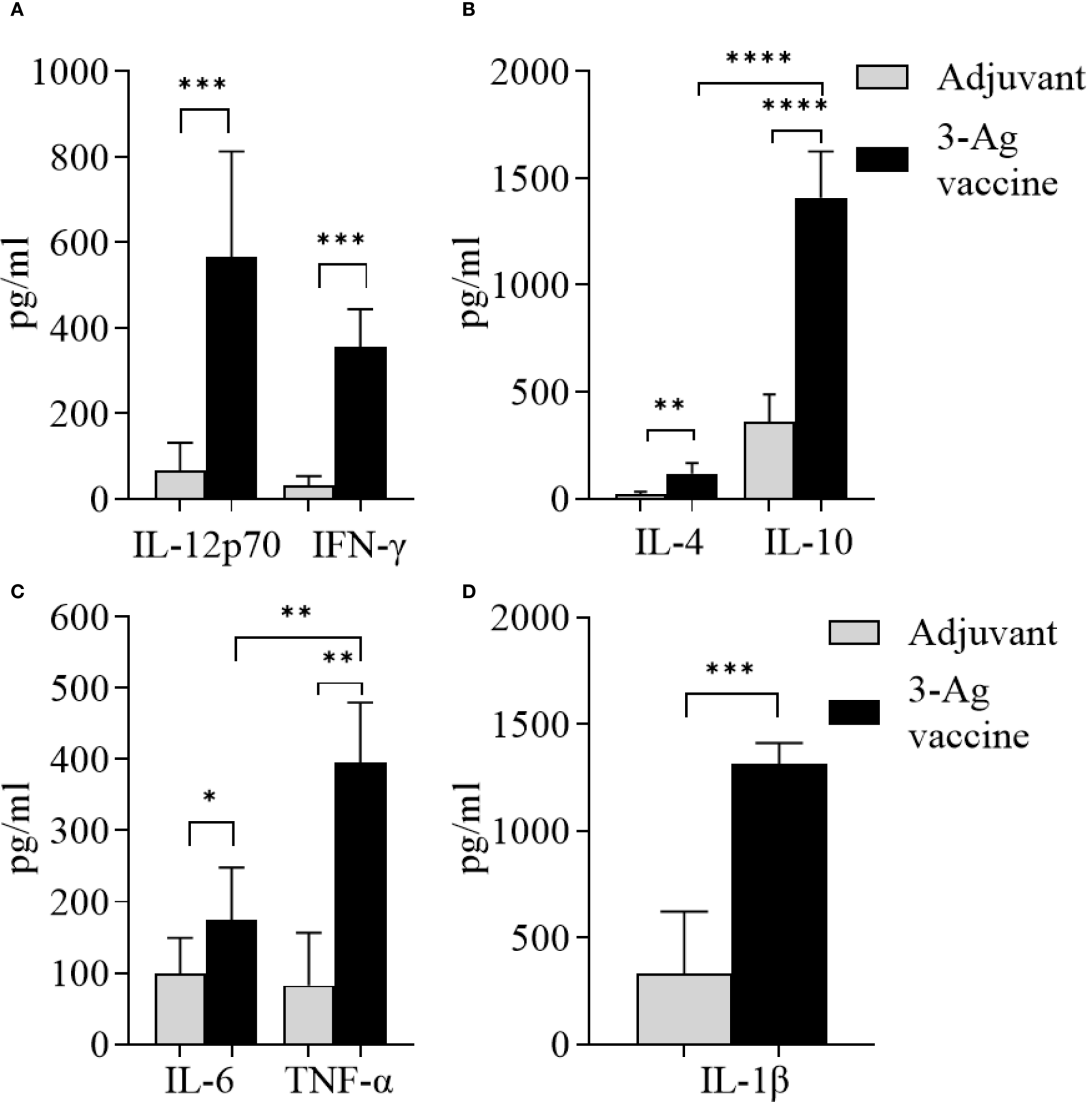
Analysis of cytokines production in sera from mice immunized with the 3-Ag vaccine. **(A)** Th1 cytokines IL-12p70 and IFN-γ, **(B)** Th2 cytokines IL-4 and IL-10, **(C)** Inflammatory cytokines IL-6 and TNF-α, and **(D)** IL-1β measured by ELISA in pooled sera from female BALB/c mice immunized with the 3-Ag vaccine (black bars) or Alum+MPLA alone (gray bars). Sera were evaluated in quadruplicate and cytokines were expressed in pg/ml ± SD. *p < 0.05; **p ≤ 0.008, ***p ≤ 0.0007, and ****p < 0.0001 by unpaired t test.

Serum cytokine levels induced by the 3-Ag vaccine were also measured by ELISA. Slightly higher IL-12p70 levels than IFN-γ (**Figure 4A**) and significantly higher IL-10 levels than IL-4 (**Figure 4B**) were detected, confirming the balanced Th1/Th2 immune response with a slight Th2 bias previously observed (37), along with the IgG subclasses profile. An increase of pro-inflammatory cytokines IL-6, TNF-α and IL-1β production was induced by the 3-Ag vaccine compared to Alum+MPLA (**Figures 4C, D**, black bars and gray bars, respectively); while these cytokines support immune activation, they can be also associated with inflammation and adverse effects (47).

### Immune responses to a low-dose 3-Ag vaccine in female and male mice

**Figure 5:**
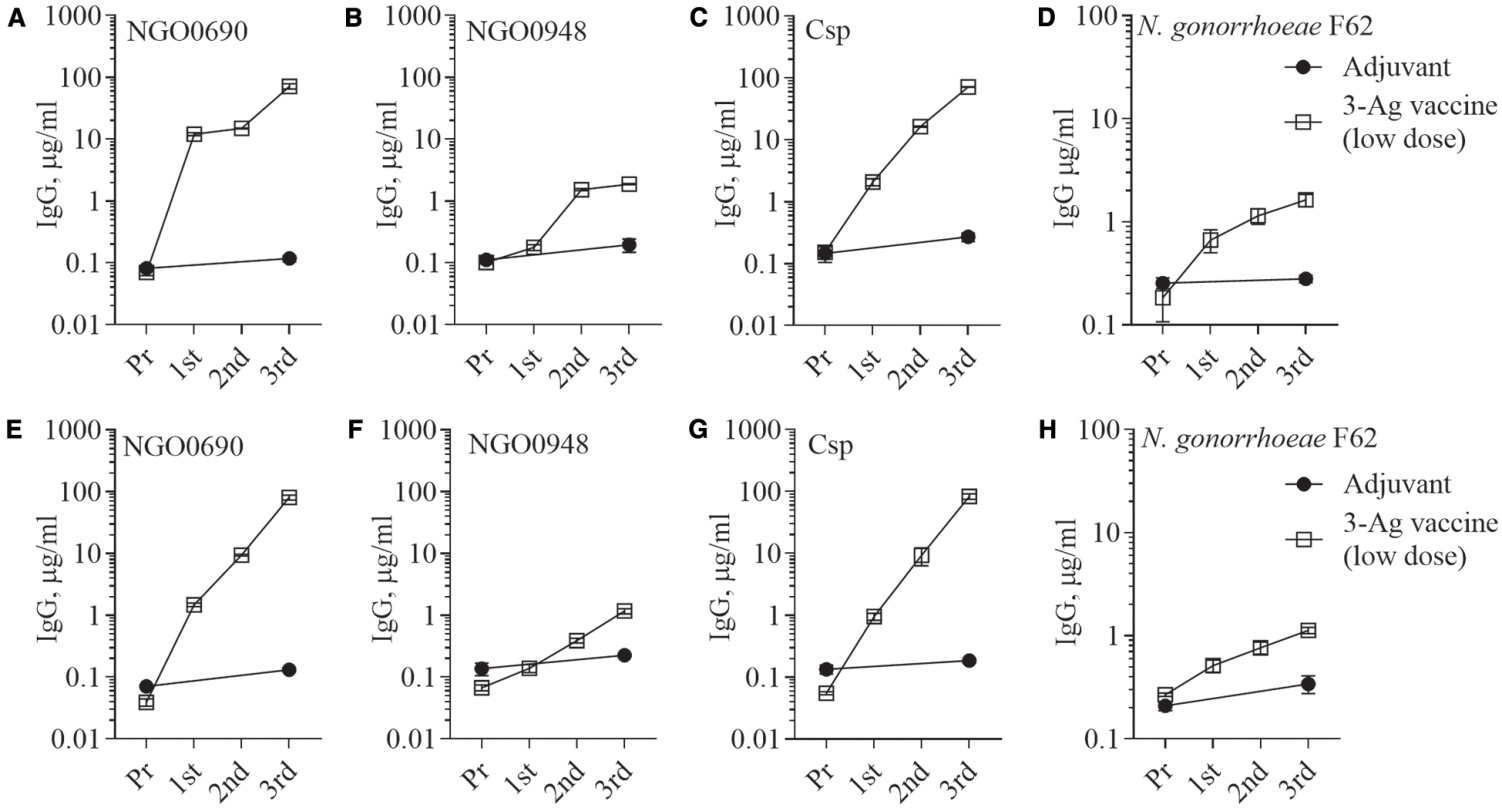
Serum IgG antibodies to the purified antigens and to *N. gonorrhoeae*. Total IgG antibodies measured by ELISA in sera from mice immunized with the low-dose 3-Ag vaccine (open squares) or Alum+MPLA alone (closed circles). Sera were evaluated against **(A)** purified recombinant NGO0690, **(B)** NGO0948 and **(C)** Csp, or **(D)** *N. gonorrhoeae* F62 in the mouse preimmune sera (Pr) and in sera collected 2 weeks after each immunization (1^st^, 2^nd,^ and 3^rd^). **(E-H)** Pooled sera from male BALB/c mice as above. Pooled sera were evaluated in triplicate and antibodies were expressed as µg/ml ± SD.

**Figure 6:**
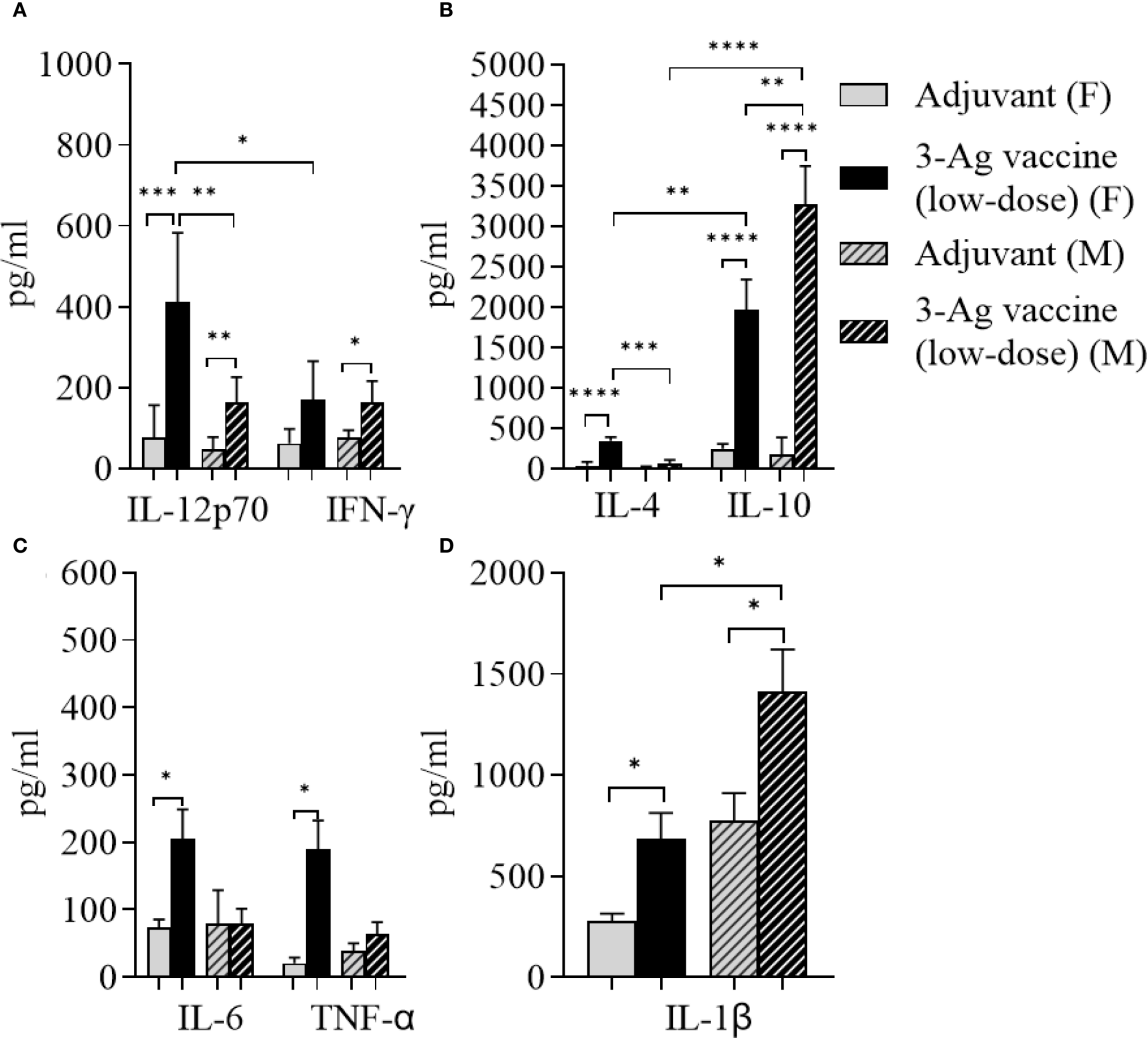
Analysis of cytokines production in sera from mice immunized with the low-dose 3-Ag vaccine in female and male mice. **(A)** Th1 cytokines IL-12p70 and IFN-γ, **(B)** Th2 cytokines IL-4 and IL-10, **(C)** Inflammatory cytokines IL-6 and TNF-α, and **(D)** IL-1β measured by ELISA in pooled sera from female BALB/c mice immunized with the low dose 3-Ag vaccine (black bars) or Alum+MPLA alone (gray bars), and pooled sera from male BALB/c mice immunized as above (gray dashed bars and black dashed bars). Sera were evaluated in quadruplicate and cytokines were expressed in pg/ml ± SD. *p < 0.05; **p ≤ 0.006, ***p ≤ 0.0005, and ****p < 0.0001 by unpaired t test.

Since the 3-Ag vaccine induced high IgG antibody levels particularly against NGO0690 and Csp, as well as production of TNF-α and IL-1β, we investigated whether antigen dose de-escalation could decrease inflammatory responses without significantly affecting antibody responses and efficacy. A low-dose 3-Ag vaccine containing 50% less of each antigen (5 µg each) but the same amount of Alum and MPLA adjuvants, was used to immunize female BALB/c mice. In addition, male mice were also immunized to begin comparing sex-dependent immune responses. No significant change in IgG levels against the individual antigens was detected in sera from the female mice compared to the original dose vaccine (**Figures 2A–C** and **5A–C**), but a decrease in IgG antibodies against *N. gonorrhoeae* F62 was observed (**Figure 5D**). In male mice, the amounts of anti-NGO0690 and anti-Csp IgG antibodies were similar to the female mice (**Figures 5E, G**), but anti-NGO0948 IgG antibodies (**Figure 5F**) and IgG antibodies against *N. gonorrhoeae* F62 (**Figure 5H**) appeared lower. In both sets of sera, slightly lower amounts of anti-gonococcal IgM antibodies were measured than with the original dose vaccine (not shown), as well as serum IgA antibodies reacting with *N. gonorrhoeae* (**Supplementary Figure S1B**, dashed and black bars, respectively). Mucosal IgA antibodies against *N. gonorrhoeae* F62 were similar to the original dose vaccine levels (**Supplementary Figure S1B**, dotted bar), while secreted IgG levels appeared lower (**Supplementary Figure S2B**). A decrease in serum IgG antibody subclasses was also determined against *N. gonorrhoeae* F62, with IgG1 = 0.77 ± 0.04 µg/ml and IgG2a = 0.52 ± 0.01 µg/ml in female mice, and IgG1 = 0.94 ± 0.07 µg/ml and IgG2a = 0.56 ± 0.005 µg/ml in male mice, but the IgG1/IgG2a ratio remained consistent with a Th1-Th2 balanced profile (Female mice, 0.68 and Male mice, 0.59). This was supported by the cytokine profile in female mice, with higher IL-12p70 than IFN-γ, and higher IL-10 than IL-4 (**Figures 6A, B**) and in male mice, although, interestingly, lower IL-12p70 and higher IL-10 were induced than in female mice (**Figures 6A, B**). The low-dose 3-Ag vaccine induced less TNF-α and IL-1β than the original dose vaccine in female mice, and no significant changes in IL-6 (**Figures 6C, D**); IL-6 and TNF-α levels were comparable to those in male mice (**Figure 6C**), but IL-1β production in male mice was higher (**Figure 6D**), even in the adjuvant-only group. These results were consistent with the reported variability in innate and adaptive immune responses to vaccination across sexes in mice and in humans (48–51).

**Figure 7:**
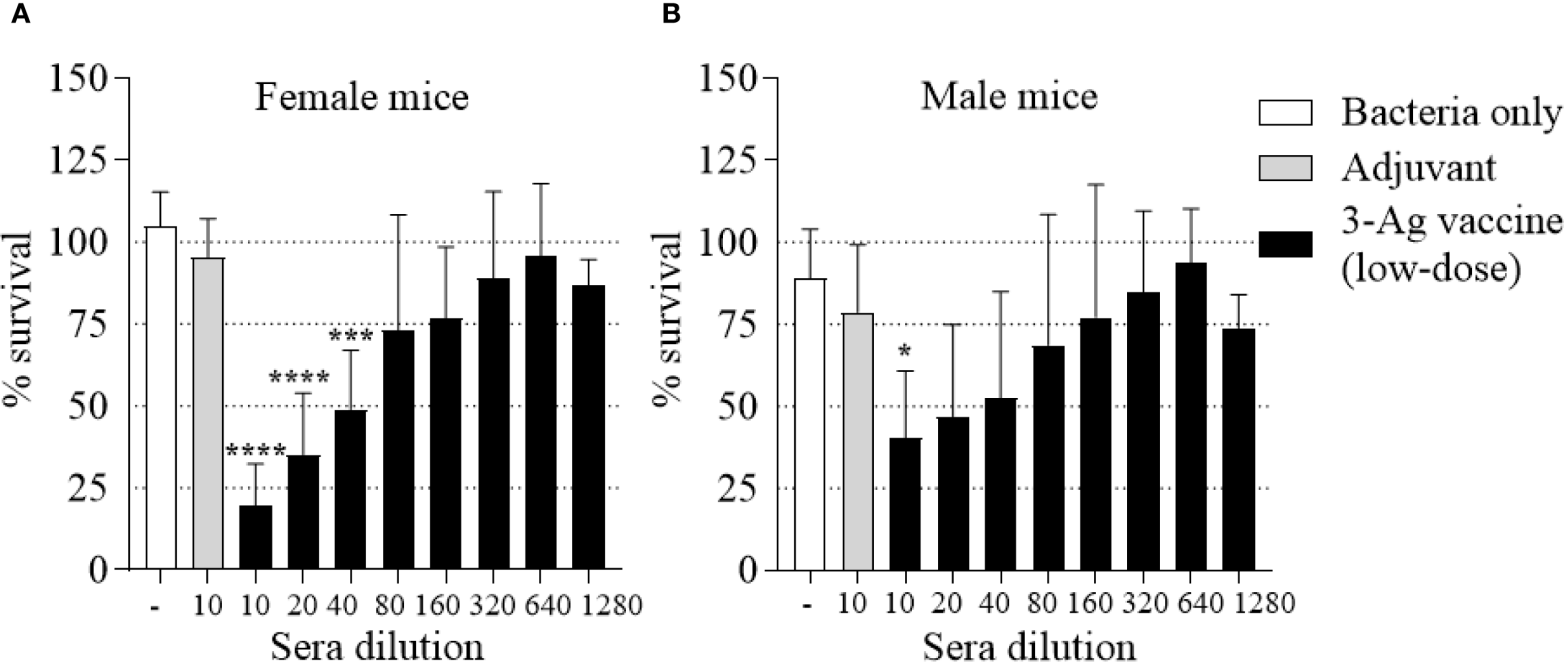
Serum Bactericidal Activity (SBA) of mouse sera elicited by immunization with the low-dose 3-Ag vaccine in female and male mice. *N. gonorrhoeae* F62 survival (% CFU at T30/T0 ± SD) in pooled sera from **(A)** Female BALB/c mice and **(B)** Male BALB/c mice immunized with the low-dose 3-Ag vaccine (black bars) or Alum+MPLA alone (gray bars). Sera were evaluated in a minimum of three repeats and dilutions are indicated on the X-axis. Bacteria alone, white bars. *p < 0.05, and ****p < 0.0001 by one-way ANOVA with Dunnett’s multiple comparisons test vs Adjuvant.

Lastly, the SBA titers were evaluated. Sera from female and male mice immunized with the low-dose vaccine retained the ability to kill *N. gonorrhoeae* F62, but an approx. 50% decrease in the SBA titers was detected in female mouse sera (**Figure 7A**) than with the original dose 3-Ag vaccine (1/80-1/160). Notably, SBA titers of male mice sera only reached ~ 1/10 (**Figure 7B**), possibly mirroring the lower magnitude of the antibody responses to *N. gonorrhoeae* detected in these mice. These results indicated that a 50% decrease in antigen amount in the 3-Ag vaccine reduced inflammatory responses but also dampened antibody responses against *N. gonorrhoeae*.

### Immunization with NGO0690+Csp and Alum+MPLA (2-Ag vaccine) also protects mice from gonococcal vaginal colonization

**Figure 8:**
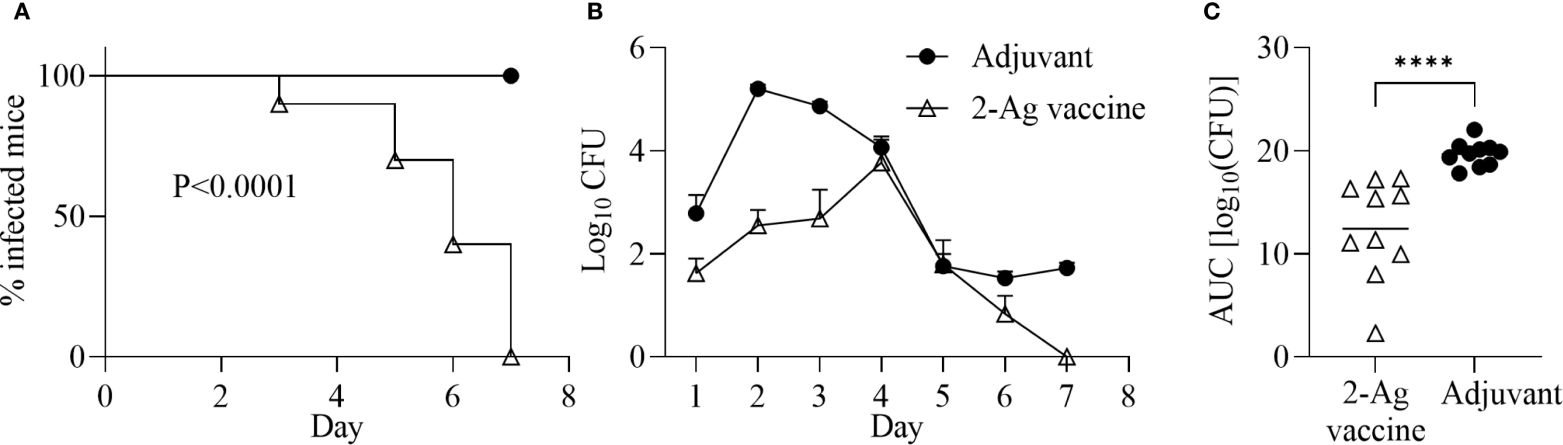
Immunization with NGO0690+Csp and Alum+MPLA (2-Ag vaccine) is protective in a mouse model of gonococcal vaginal colonization. Female BALB/c mice (n = 20) were immunized with the 2-Ag vaccine (10 µg each antigen) (open triangles) or with adjuvants alone (black circles). Two weeks after the third immunization, mice in the diestrus phase of the estrous cycle (n = 10 per group) were challenged intravaginally with *N. gonorrhoeae* FA1090 (3.1 x 10^7^ CFU). Vaginal swabs were collected daily and plated to enumerate gonococcal CFUs. **(A)** Time-to-clearance Kaplan–Meier curves (p < 0.0001, groups compared by Mantel-Cox analysis); **(B)** Bacterial burden (log_10_ CFU ± SEM); **(C)** Area under the curve (AUC) (means ± 95% confidence intervals compared across groups by Mann Whitney’s non-parametric test). ****p < 0.0001).

**Figure 9:**
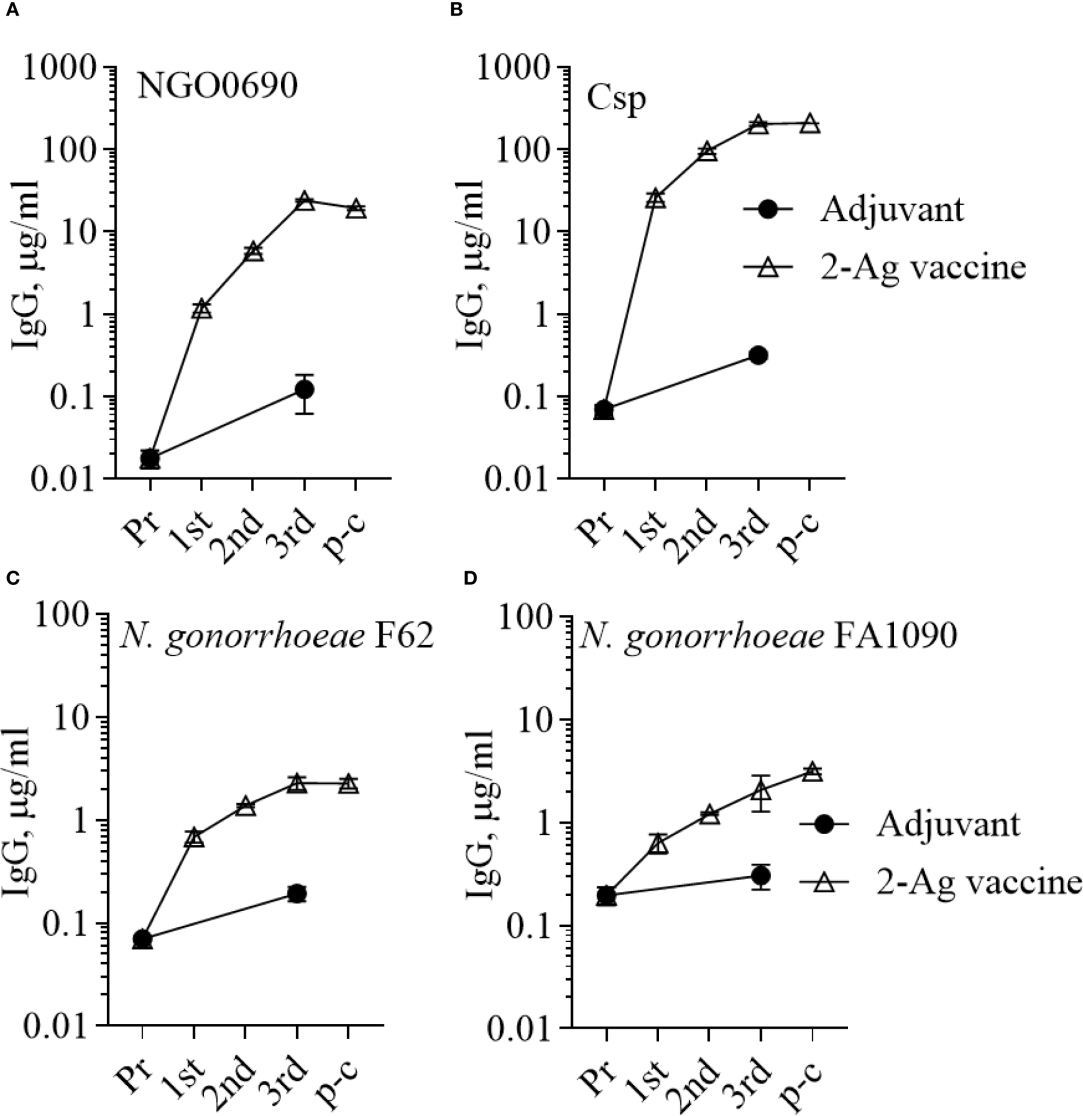
Serum IgG antibodies to the purified antigens and to *N. gonorrhoeae.* Total IgG antibodies measured by ELISA in pooled sera from mice immunized with the 2-Ag vaccine (open triangles) or with Alum+MPLA alone (closed circles) against **(A)** NGO0690, **(B)** Csp, **(C)** *N. gonorrhoeae* F62 and **(D)** *N. gonorrhoeae* FA1090. Pr, preimmune; p-c, pre-challenge. Sera were evaluated in triplicate and antibodies were expressed as µg/ml ± SD.

**Figure 10:**
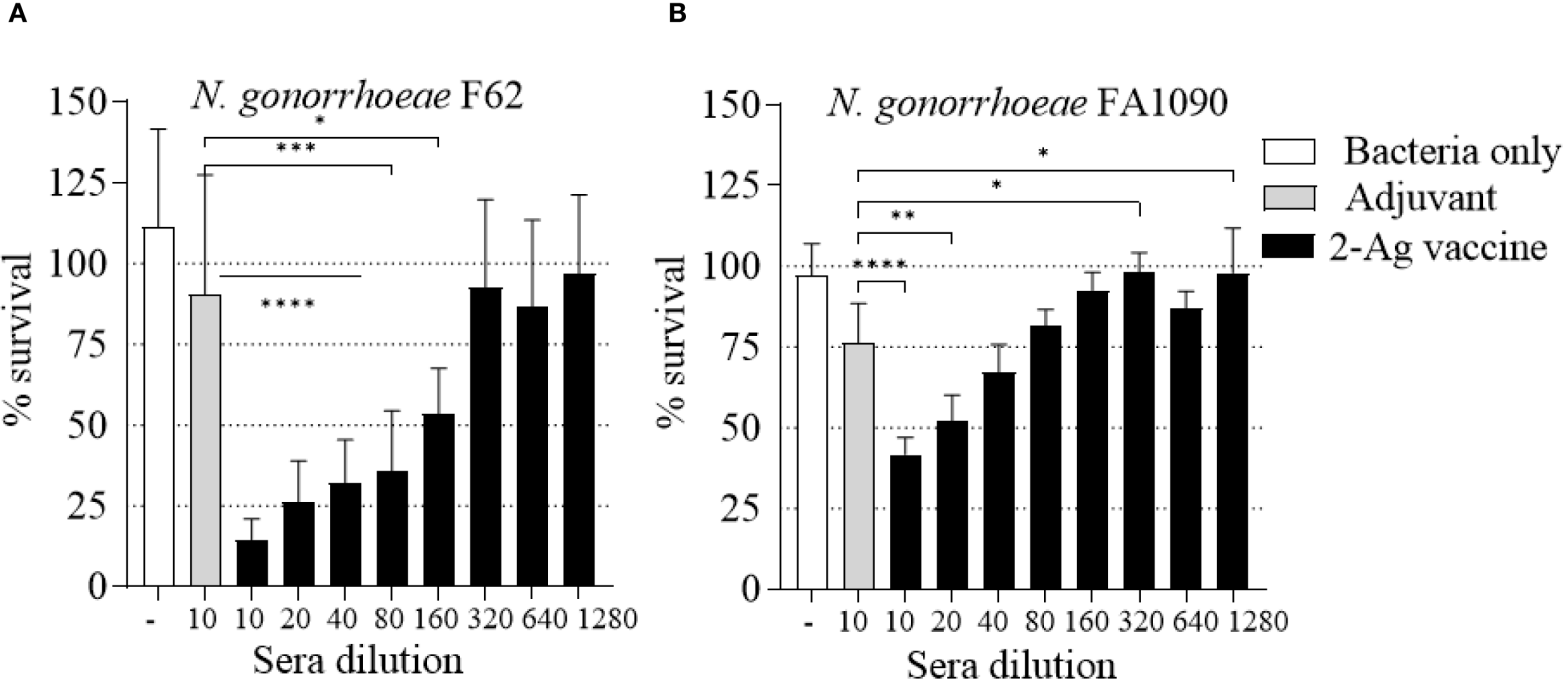
Serum Bactericidal Activity (SBA) of mouse sera elicited by immunization with the 2-Ag vaccine. *N. gonorrhoeae* survival (% CFU at T30/T0 ± SD). Pooled sera from female BALB/c mice immunized with the 2-Ag vaccine (black bars) or Alum+MPLA alone (gray bars). **(A)** *N. gonorrhoeae* F62 and **(B)** *N. gonorrhoeae* FA1090. Bacteria alone, white bars. Sera were evaluated in a minimum of three repeats and dilutions are indicated on the X-axis. *p ≤ 0.03, **p <= 0.005, ***p = 0.0001 and ****p < 0.0001 by one-way ANOVA with Dunnett’s multiple comparisons test vs Adjuvant.

Since NGO0690 and Csp were more immunodominant than NGO0948, and because the latter is a structurally more complex antigen that could be more difficult to scale up for manufacturing, we evaluated a bivalent vaccine only comprising NGO0690 and Csp. Female BALB/c mice were immunized with NGO0690+Csp (10 µg each) and Alum+MPLA as adjuvants (2-Ag vaccine) followed by challenge with *N. gonorrhoeae* FA1090 as previously described. The 2-Ag vaccine also protected mice when compared to the Alum+MPLA control group (**Figures 8A–C**), indicating induction of protective responses *in vivo*. Qualitative, quantitative, and functional antibody responses elicited by the 2-Ag vaccine were examined; as expected, anti-NGO0690 and anti-Csp IgG antibodies remained high (**Figure 9A**), and only a slight decrease in IgG levels against *N. gonorrhoeae* was observed (**Figure 9B**). Comparable IgM antibody responses were reported (not shown) and serum IgA antibodies (**Supplementary Figure S1C**, striped bars), as well as mucosal IgA against *N. gonorrhoeae* F62 (**Supplementary Figure S2C**, dotted bars) but an apparent decrease in mucosal IgA against *N. gonorrhoeae* FA1090. IgG levels in vaginal lavages remained high (**Supplementary Figure S2C**). The observed balanced Th1/Th2 response was also confirmed by evaluation of the IgG subclasses induced by the 2-Ag vaccine, with IgG1 = 1.19 ± 0.06 µg/ml and 1.67 ± 0.05 µg/ml for *N. gonorrhoeae* F62 and FA1090, respectively, and IgG2a: 0.7 ± 0.08 µg/ml for both strains. The Th1 and Th2 cytokines profile was quantitatively and qualitatively similar to that of the 3-Ag vaccine (not shown), including that of IL-6 and IL-1β. However, an approx. 50% reduction in TNF-α levels was observed (185 ± 31 pg/ml vs 395 ± 84 pg/ml for the 3-Ag vaccine) suggesting that NGO0948 contributed to inflammatory responses. The SBA titers of the 2-Ag vaccine sera also remained around 1/80 - 1/160 against *N. gonorrhoeae* F62 (**Figure 10A**) but were only about 1/10 - 1/20 against *N. gonorrhoeae* FA1090 (**Figure 10B**). These results indicated that NGO0690 and Csp were immunodominant antigens that induced majority of the antibody responses against *N. gonorrhoeae* and suggested that, while exclusion of NGO0948 only minimally affected antibody production *in vivo*, it decreased inflammatory responses.

## Discussion

While *N. gonorrhoeae* whole-bacteria vaccines have failed to induce cross-strain protection because of antigen variability, they have served as a stepping-stone for identification of potential gonococcal targets for use in subunit vaccines (12). For some diseases, monovalent subunit vaccines may be suitable [for example, the *H. influenzae* type B (Hib) vaccine conjugate, the shingles vaccine, the Hepatitis B (HepB) vaccines or Covid-19 (52)], but a multivalent vaccine may offer significant advantages against a pathogen such as *Neisseria gonorrhoeae*, where antigenic variability and immune evasion strategies are a concern (12) (ideally, immune responses against multiple conserved antigens that target distinct bacterial functions will reduce the possibility of vaccine escape). From an immunological standpoint, multivalent vaccines may also enhance the magnitude or the quality of the immune response because of potential synergistic effects among the immunodominant and conserved antigens used (purified proteins, polysaccharides, or peptides) (38). Gonococcal proteins that have been tested in combination include LptD and LtgC (53), the passenger and translocator fragments of adhesion and penetration protein (App) (54), TbpA and TbpB (55), App, MetQ and NHBA (56), NGO0265 and NGO1549 (57), which have indeed shown more robust immune responses and sustained bactericidal titers than the individual antigens. Our previous studies of NGO0690, NGO0948 and Csp as a combination vaccine aligned with these observations (33, 37). Building on those results, we assessed the ability of the 3-Ag vaccine to induce protective responses in a mouse model of gonococcal vaginal colonization. Our results showed that the 3-Ag vaccine induced protection against *N. gonorrhoeae in vivo*, demonstrated by a reduced bacterial burden and an accelerated colonization clearance time compared to the adjuvant control group. We confirmed that the 3-Ag vaccine elicited strong systemic antibody responses against the individual antigens and against whole gonococci, along with mucosal IgG and low levels of mucosal IgA antibodies. Mucosal responses could be enhanced by exploring different adjuvants and/or routes of immunization in future studies (27, 37, 56, 58) including, for example, systemic priming followed by mucosal boost, as shown against Chlamydia (59, 60) and in other infection models (61). The bactericidal activity of sera from the vaccinated mice against *N. gonorrhoeae* strains showed titers aligning with those we previously reported, thus reinforcing the value of our vaccine targets. Based on the IgG subclasses and on the serum cytokine profile elicited, a balanced Th1/Th2 response was observed, marked primarily by IL-12p70 and IL-10. These results suggested a likely multifactorial immune mechanism involving both humoral and cellular components promoting a broad-based immune response that contributed to both bacterial clearance and antibody functionality. Detectable serum levels of proinflammatory cytokines TNF-α and IL-1β further supported immune system activation, but also suggested potential vaccine-induced inflammation (62).

For this reason, we investigated whether de-escalating the antigens dose could offer immunological advantages, such as minimizing potential inflammation without affecting functional antibody responses. In some cases, decreasing the antigen concentration may even improve T cell response and induction of memory B cells crucial for long-term immunity (63) although, if the antigen concentration is too low, the immune system might not be sufficiently stimulated to generate a robust and long-lasting protective response. Fine-tuning of the antigens dose in a multivalent vaccine is also important to avoid potential immune interference among the antigens (63). We reported no significant changes in the quantity and quality of the antibody responses to the purified proteins in sera from mice that were immunized with a 3-Ag vaccine that contained 50% less of each antigen, but antibody titers elicited by the low-dose vaccine against whole gonococci appeared lower. While it is possible that the immunoreactive epitopes are more accessible to the antibodies on the purified antigens than when these are expressed within the bacterial membrane, this finding realistically reflected the antigen dose-dependency of the immune responses induced.

Qualitatively, the antibody responses were similar in female and male mice, but a slightly dampened profile was observed in male mice, with lower antibody levels against *N. gonorrhoeae* and lower SBA titers. The serum cytokine profile was also different in male mice, with more elevated IL-10 and reduced IL-12p70, TNF-α and IL-6 than in female mice, and a higher IL-1β baseline. These results align with a more robust humoral and cell-mediated response often detected in females than in males, which is attributed to hormonal, genetic, or microbiota-driven factors increasingly recognized as key modulators of vaccine efficacy (48, 50, 51). These findings underscore the importance of including both sexes in preclinical vaccine evaluation, since differences in immune responses between sexes may affect translational outcomes in humans, especially for gonococcal-specific immune responses. Indeed, vaccine efficacy studies have suggested that, for certain vaccines, one sex may be better protected than the other, for example measles vaccination, the seasonal trivalent influenza vaccine or hepatitis B seem more effective in females, and BCG vaccination may be more effective in males, and thus requiring more fine-tuning (48–50).

Based on the strong antibody responses induced by NGO0690 and Csp, and on the higher SBA titers induced by the individual antigens previously reported (33), these candidates may be more immunodominant targets compared to NGO0948. We adopted an immunofocusing strategy by excluding NGO0948, an approach that could also be beneficial from a potential future manufacturing perspective, since NGO0948 is a large and structurally more complex protein. The NGO0690+Csp bivalent formulation was found to also protect mice *in vivo*, although the apparent longer time to infection clearance and the lower SBA titers measured than with the 3-Ag vaccine warrant additional investigation. However, the inherent variability across experiments in the mouse model makes quantitative comparisons between experiments problematic. It is possible that the breadth and magnitude of protection conferred by the 3-Ag combination was likely superior, despite an apparently more modest contribution of NGO0948 to the antibody responses. On the other hand, using exclusively immunodominant antigens in a vaccine may not always be beneficial, since it may lead to immune evasion or even induction of blocking antibodies, as with gonococcal Rmp protein (64). Thus, presence of sub-dominant antigens could be a strength for broadening the response to a multi-antigen vaccine (41, 65, 66); we cannot exclude the possibility that additional CASS antigens may be added to NGO0690 and Csp in a multivalent vaccine to achieve similar, or even better protection. The benefits of adding additional antigens to a vaccine preparation need to be weighed against increasing costs, an important consideration for this disease that disproportionately affects socioeconomically disadvantaged populations and persons in low- and middle-income countries (LMICs). Altogether, this work adds to a growing body of evidence supporting subunit-based vaccination strategies for gonorrhea because these may offer customizable approaches, particularly when optimized for conserved surface-exposed gonococcal antigens. In addition, different adjuvants should be explored to increase the effectiveness of our antigens, or different delivery platforms targeting mucosal tissues, for improving mucosal antibodies and memory responses critical for protection against gonorrhea. Alternatively, OMVs could be engineered in which CASS antigens are overexpressed, to benefit from the built-in adjuvanticity, ease of purification and flexibility of this platform. Besides antigen composition, future studies should also continue to focus on refining antigen doses and defining the immune correlates of protection across sexes.

## Data Availability

The raw data supporting the conclusions of this article will be made available by the authors, without undue reservation.
