# Supplementary material for: Preclinical in vivo evaluation of a gonococcal multivalent vaccine containing antigens identified by CASS
Source: Front Immunol. 2025 Sep 22;16:1688536. doi: 10.3389/fimmu.2025.1688536 (PMC12497722; doi:10.3389/fimmu.2025.1688536)
Supplement: Supplementary file 1 [file Presentation1.pptx]

## Slide 1
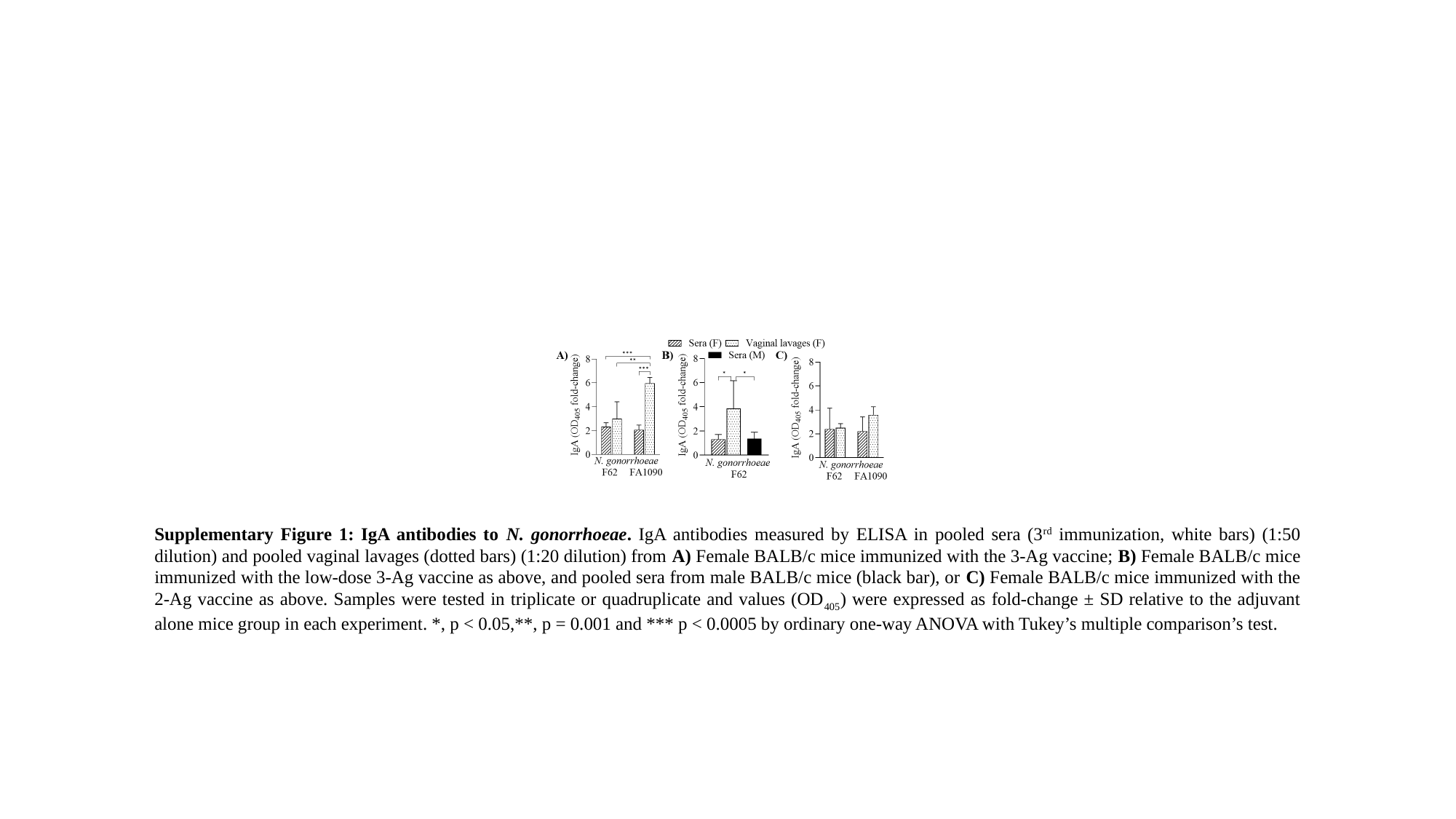

Supplementary Figure 1: IgA antibodies to N. gonorrhoeae. IgA antibodies measured by ELISA in pooled sera (3rd immunization, white bars) (1:50 dilution) and pooled vaginal lavages (dotted bars) (1:20 dilution) from A) Female BALB/c mice immunized with the 3-Ag vaccine; B) Female BALB/c mice immunized with the low-dose 3-Ag vaccine as above, and pooled sera from male BALB/c mice (black bar), or C) Female BALB/c mice immunized with the 2-Ag vaccine as above. Samples were tested in triplicate or quadruplicate and values (OD405) were expressed as fold-change ± SD relative to the adjuvant alone mice group in each experiment. *, p < 0.05,**, p = 0.001 and *** p < 0.0005 by ordinary one-way ANOVA with Tukey’s multiple comparison’s test.
