# Supplementary material for: Preclinical in vivo evaluation of a gonococcal multivalent vaccine containing antigens identified by CASS
Source: Front Immunol. 2025 Sep 22;16:1688536. doi: 10.3389/fimmu.2025.1688536 (PMC12497722; doi:10.3389/fimmu.2025.1688536)
Supplement: Supplementary file 2 [file Presentation2.pptx]

## Slide 1
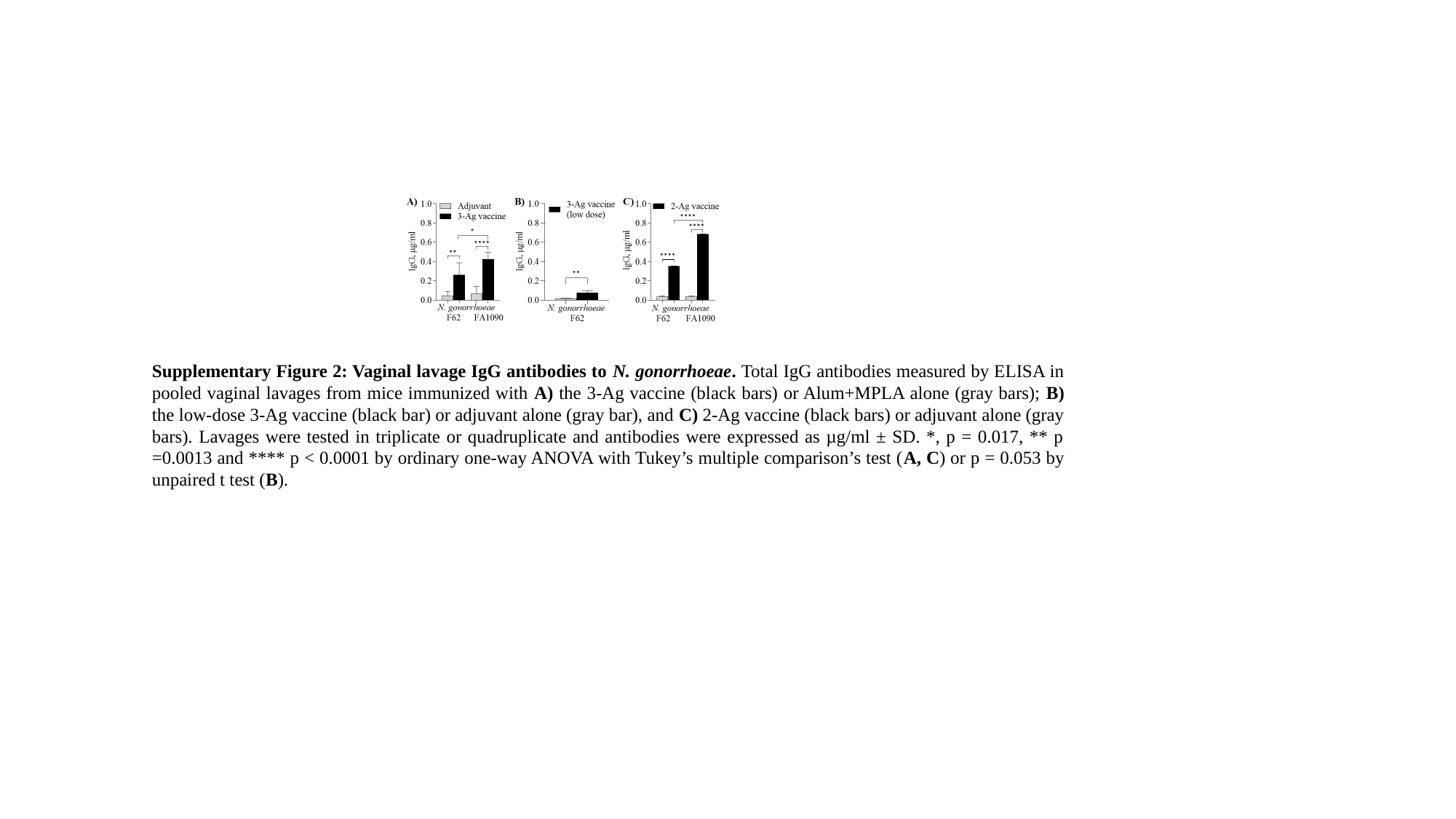

Supplementary Figure 2: Vaginal lavage IgG antibodies to N. gonorrhoeae. Total IgG antibodies measured by ELISA in pooled vaginal lavages from mice immunized with A) the 3-Ag vaccine (black bars) or Alum+MPLA alone (gray bars); B) the low-dose 3-Ag vaccine (black bar) or adjuvant alone (gray bar), and C) 2-Ag vaccine (black bars) or adjuvant alone (gray bars). Lavages were tested in triplicate or quadruplicate and antibodies were expressed as µg/ml ± SD. *, p = 0.017, ** p =0.0013 and **** p < 0.0001 by ordinary one-way ANOVA with Tukey’s multiple comparison’s test (A, C) or p = 0.053 by unpaired t test (B).
