# Supplementary material for: Preclinical in vivo evaluation of a gonococcal multivalent vaccine containing antigens identified by CASS
Source: Front Immunol. 2025 Sep 22;16:1688536. doi: 10.3389/fimmu.2025.1688536 (PMC12497722; doi:10.3389/fimmu.2025.1688536)
Supplement: Supplementary file 3 [file Table1.docx]

| **Supplementary Table 1: Serum IgG subclasses against *N. gonorrhoeae*** | | | |
| --- | --- | --- | --- |
| ***N. gonorrhoeae* strain** | **IgG1^§^** | **IgG2a^§^** | **Th1/Th2 ratio^§§^** |
| **F62** | 1.2 ± 0.03 | 0.92 ± 0.04 | 0.76 |
| **FA1090** | 0.81 ±0.008 | 0.66 ± 0.05 | 0.82 |
| ^§^ µg/ml ± SD; ^§§^ IgG2a/IgG1 | | | |
